# Succinate dehydrogenase deficiency in a *PDGFRA* mutated GIST

**DOI:** 10.1186/s12885-017-3499-7

**Published:** 2017-08-02

**Authors:** Martin G. Belinsky, Kathy Q. Cai, Yan Zhou, Biao Luo, Jianming Pei, Lori Rink, Margaret von Mehren

**Affiliations:** 1 Molecular Therapeutics Program, Fox Chase Cancer Center, 333 Cottman Avenue, Philadelphia, PA 19111-2497 USA; 2 Cancer Biology Program, Fox Chase Cancer Center, Philadelphia, PA USA; 3 Biostatistics and Bioinformatics Facility, Fox Chase Cancer Center, Philadelphia, PA USA; 4 Molecular Diagnostics Laboratory, Fox Chase Cancer Center, Philadelphia, PA USA; 5 Genomics Services, Fox Chase Cancer Center, Philadelphia, PA USA

**Keywords:** Gastrointestinal stromal tumor, Kit, Platelet derived growth factor receptor alpha, Succinate dehydrogenase, Imatinib mesylate, Crenolanib

## Abstract

**Background:**

Most gastrointestinal stromal tumors (GISTs) harbor mutually exclusive gain of function mutations in the receptor tyrosine kinase (RTK) KIT (70–80%) or in the related receptor PDGFRA (~10%). These GISTs generally respond well to therapy with the RTK inhibitor imatinib mesylate (IM), although initial response is genotype-dependent. An alternate mechanism leading to GIST oncogenesis is deficiency in the succinate dehydrogenase (SDH) enzyme complex resulting from genetic or epigenetic inactivation of one of the four *SDH* subunit genes (*SDHA*, *SDHB*, *SDHC*, *SDHD*, collectively referred to as *SDHX*). SDH loss of function is generally seen only in GIST lacking RTK mutations, and SDH-deficient GIST respond poorly to imatinib therapy.

**Methods:**

Tumor and normal DNA from a GIST case carrying the IM-resistant PDGFRA D842V mutation was analyzed by whole exome sequencing (WES) to identify additional potential targets for therapy. The tumors analyzed were separate recurrences following progression on imatinib, sunitinib, and the experimental PDGFRA inhibitor crenolanib. Tumor sections from the GIST case and a panel of ~75 additional GISTs were subjected to immunohistochemistry (IHC) for the SDHB subunit.

**Results:**

Surprisingly, a somatic, loss of function mutation in exon 4 of the *SDHB* subunit gene (c.291_292delCT, p.I97Mfs*21) was identified in both tumors. Sanger sequencing confirmed the presence of this inactivating mutation, and IHC for the SDHB subunit demonstrated that these tumors were SDH-deficient. IHC for the SDHB subunit across a panel of ~75 GIST cases failed to detect SDH deficiency in other GISTs with RTK mutations.

**Conclusions:**

This is the first reported case of a *PDGFRA* mutant GIST exhibiting SDH-deficiency. A brief discussion of the relevant GIST literature is included.

**Electronic supplementary material:**

The online version of this article (doi:10.1186/s12885-017-3499-7) contains supplementary material, which is available to authorized users.

## Background

Gastrointestinal stromal tumor, or GIST, is the most common mesenchymal tumor of the GI tract, with an estimated annual incidence of 14–20 cases per million [1]. GIST generally presents as a sporadic disease in older adults (median age 60–65 years), and affects men and women equally. GIST may originate throughout the GI tract but occurs most commonly in the stomach (~60%) or small intestine (~25%) [2]. These tumors are related to spindle-shaped pacemaker cells of the gut known as the interstitial cells of Cajal (ICC) with which they share phenotypic characteristics including the expression of the type III receptor tyrosine kinase (RTK) KIT (CD117) [3, 4]. Mutually exclusive gain of function mutations in KIT, or in the related RTK PDGFRA, are found in ~85% of GIST, and lead to increased kinase signaling including MAPK and PI3K-AKT [5, 6]. The discovery of activating mutations in these receptors in GIST led to the approval of the RTK inhibitor imatinib mesylate (IM) as front-line therapy for GIST, and subsequent approval of sunitinib malate and regorafenib for IM-resistant disease. While most RTK-mutated GISTs are IM-sensitive, therapeutic response is influenced by genotype. Among the most common mutation types, GISTs with mutations in the juxtamembrane domain encoded by *KIT* exon 11 are generally IM-sensitive while GISTs harboring *KIT* exon 9 mutations require IM dose-escalation. The most common *PDGFRA* mutation, the D842V mutation in exon 18 encoding the kinase activation loop, confers primary clinical resistance to IM [7, 8].

The 10–15% of GIST that lack mutations in *KIT* and *PDGFRA,* known as wild type or, more accurately, RTK-wild type GIST, also respond poorly to IM therapy [9, 10]. RTK-wild type GIST may harbor mutations in genes that activate kinase signaling downstream of the receptors, particularly in the RAS/RAF/MAPK pathway. Germline loss of function mutations in neurofibromin, a RAS-GAP that acts as a negative regulator of RAS signaling, are linked to neurofibromatosis type 1 (NF1), and predispose NF1 individuals to development of a variety of tumor types including GIST. While NF1-related GIST has been reported to comprise ~1.5% of the patient population of GIST [11], reports of sporadic or constitutional *NF1* gene mutations in undiagnosed NF1 individuals suggests this percentage may be somewhat higher [12, 13]. The gain of function V600E mutation in the *BRAF* gene has been found in ~7% of GISTs lacking *KIT/PDGFRA* mutations [14, 15], while a small number of cases with activating mutations in the *KRAS* gene have also been reported [16]. There is limited clinical data on the efficacy of IM or other RTK inhibitors in RAS pathway mutated GIST [2].

A distinct mechanism of oncogenesis seen in approximately 40% of RTK-wild type GIST is deficiency in the mitochondrially located tumor suppressor complex succinate dehydrogenase (SDH). SDH is a heterotetrameric enzyme complex that connects the oxidation of succinate to fumarate in the Krebs cycle to the reduction of coenzyme Q in the mitochondrial electron transport chain. Mutation or silencing of any of the four *SDH* genes (*SDHA-D*, or collectively *SDHX*) destabilizes the complex and results in accumulation of succinate and activation of cellular pathways that lead to increased angiogenesis and cellular proliferation [17]. SDH-deficient GIST includes GIST that occur in association with rare multitumor syndromes, the majority of GIST that occur in children, and a subset of sporadic adult gastric GISTs. The Carney-Stratakis syndrome (CSS), an inherited multi-tumor syndrome characterized by the occurrence of multifocal gastric GIST and multicentric paraganglioma (PGL) [18], is caused by germline mutations in the *SDHB*, *SDHC*, or *SDHD* subunit genes [19, 20]. The Carney triad (CT) is a non-familial association of gastric GIST, PGL and pulmonary chondromas that occurs primarily in young females [21]. CT and most pediatric GIST lack *SDHX* mutations but exhibit SDH deficiency due to epigenetic silencing of the *SDHC* gene through promoter hyper-methylation [22–25]. More recently, mutations in the *SDHA* gene subunit have been identified in a subset of sporadic adult, RTK-wild type gastric GISTs [26–31]. Analysis of large GIST sample sets has established that SDH deficiency is largely mutually exclusive to *KIT*/*PDGFRA*/*BRAF*/*NF1* mutation [23, 32, 33].

Here we report whole exome sequencing (WES) analysis of two GISTs harboring the IM-insensitive PDGFRA D842V mutation. These tumors, resected at different times from the same patient, were found to harbor an inactivating mutation in the *SDHB* gene in addition to the *PDGFRA* mutation. Immunohistochemical (IHC) analysis demonstrated complete lack of SDHB expression in the GISTs, confirming SDH-deficiency. This report describes for the first time the co-existence of *PDGFRA* and *SDH* gene mutations in a GIST case. A brief review of the literature describing the co-occurrence of mutations in *PDGFRA* or *KIT* and *SDH* subunit genes follows.

## Methods

### Sequence analysis of patient tumor samples

The Fox Chase Biosample Repository (BSR) obtains, deposits and maintains patient samples following informed written consent. De-identified patient pathological and molecular reports available from the BSR were queried for GIST samples with mutations in *PDGFRA*. Tumor samples and normal blood from four patients with the IM-resistant D842V mutation were obtained from the BSR under a protocol approved by the Fox Chase Cancer Center Institutional Review Board (#03–848). Additional associated de-identified clinical data from the case in question were obtained from the BSR data warehouse that contains demographic data, clinical information and treatment outcomes. The isolation and characterization of genomic DNA for whole-exome sequencing (WES) from frozen tumor specimens has been described [12]. Exome-enriched genomic libraries (Sureselect human all exon V4, Agilent Technologies, Santa Clara, CA) from normal and tumor DNA were subjected to paired-end 100 bp sequencing on the Illumina HiSeq 2000 instrument (Illumina, San Diego, CA). Reads were mapped to the reference human genome (Hg19 corresponding v37) using the BWA aligner [34] and mapped reads were sorted, merged, and de-duplicated (Picard). Local realignments were done using GATK in areas surrounding insertions and deletions (indels) [35, 36]. Variant calling was performed using GATK UnifiedGenotyper [35, 36] and single nucleotide variants (SNVs) annotation and variant effect predictions were done with ANNOVAR [37] by querying various databases. Non-synonymous, potentially deleterious coding region variants, splice-site mutations, and insertions or deletions (indels) that were predicted to be present in the tumor only, were visually confirmed on the Integrative Genomics Viewer (IGV) [38], and confirmed by exon-based Sanger sequencing. Primer sequences for confirmation of mutations listed in Table 1 are shown in Additional file 1. Relevant exons were PCR-amplified from genomic DNA and subjected to Sanger sequencing by the Fox Chase Cancer Center DNA Sequencing Facility.

**Table 1:** Confirmed deleterious^a^ or truncating somatic variants in GIST1 and GIST2

Gene symbol	UniProt accession^b^	Genomic coordinate^c^	Exon	Mutation (cDNA)	Mutation (protein)	MAF^d^ (%), (GIST 1;GIST2)
*PDGFRA*	P16234	chr4:55,152,093	18	c.2525A > T	p.D842V	33; 48
*SDHB*	P21912	chr1:17,355,226	4	c.291_292delCT	p.I97Mfs*21	42; 61
*CERS2*	Q96G23	chr1:150,939,337	9	c.743C > G	p.S248*	36; 45
*DNAH3*	Q8TD57	chr16:21,049,229	34	c.4804G > A	p.G1602S	76; 86
*CAPN9*	O14815	chr1:230,928,629	16	c.1825G > A	p.G609S	32; 32
*DIS3*	Q9Y2L1	chr13:73,347,835	8	c.1226C > T	p.S409F	17; 39
*GJD2*	Q9UKL4	chr15:35,044,921	2	c.724 T > C	p.C242R	32; 19
*EDN3*	P14138	chr20:57,876,697	2	c.285G > T	p.R95S	41; 42
*PI4KA*	P42356	chr22:21,104,246	28	c.3190A > G	p.I1064V	43; 29
*TENM2*	Q9NT68	chr5:167,645,590	23	c.4667 T > A	p.I1556N	60; 64
*JPH1*	Q9HDC5	chr8:75,227,712	3	c.523G > A	p.V175 M	25; 40
*DIS3L2*	Q8IYB7	chr2:232,880,352	2	c.181G > T	p.E61*	0; 89
*SENP6*	Q9GZR1	chr6:76,369,037	7	c.610A > G	p.K204E	0; 58
*PLCG2*	P16885	chr16:81,944,244	18	c.1853G > A	p.R618H	0; 24

^a^http://www.mypeg.info; ^b^http://www.uniprot.org; ^c^Hg19; ^d^mutant allele frequency

The symbol "*" in a description of a protein variant is standard nomenclature that denotes a stop codon in the protein sequence

### SNP array analysis

SNP array analysis was performed using Affymetrix CytoScan HD arrays (Santa Clara, California, USA). Genomic DNA was digested with *Nsp*I restriction enzyme, adaptor-ligated, and amplified using a primer recognizing the adapter sequence. Amplification products were purified using magnetic beads, fragmented, biotin-labeled, and hybridized to arrays according to the manufacturer’s recommendations. The hybridized array was washed, scanned with a GeneChip Scanner 3000 7G, and intensities of probe hybridization were analyzed using Affymetrix GeneChip Command Console. Copy number and genotyping analyses were performed using Affymetrix Chromosome Analysis Suite software with default settings. Whole genome SNP copy number data were visualized using the Affymetrix Chromosome Analysis Suite.

### Immunohistochemical analysis

IHC for CD117 (KIT) was performed as described [39]. DOG1 IHC was performed by the Fox Chase Cancer Center Clinical Pathology Laboratory using an antibody from Cell Marque (Sigma Aldrich, St. Louis, MO, USA). IHC for SDHB, and criteria used for assessing SDHB protein expression have been previously described [26, 40]. The construction of tissue microarrays (TMAs) containing ~75 clinically annotated GIST specimens has been previously described [12]. TMAs and whole tissue sections were evaluated for punctate cytoplasmic staining for SDHB. Lack of SDHB staining in GIST cells was considered informative only if typical granular cytoplasmic staining (a mitochondrial pattern) was seen in internal controls such as endothelial cells. IHC for CD31 was performed using the anti-CD31 antibody M083, clone JC70A (Dako N.A., Carpenteria, CA, USA).

### Literature search strategy

Combinations of the search terms “GIST or gastrointestinal stromal tumor” and “KIT or PDGFRA” and “SDH or succinate dehydrogenase” were used to generate a reference list of ~100 reports at the time of manuscript preparation. Abstracts from the search were individually examined for relevance and relevant studies were reviewed for the Discussion section.

## Results

**Fig. 1:**
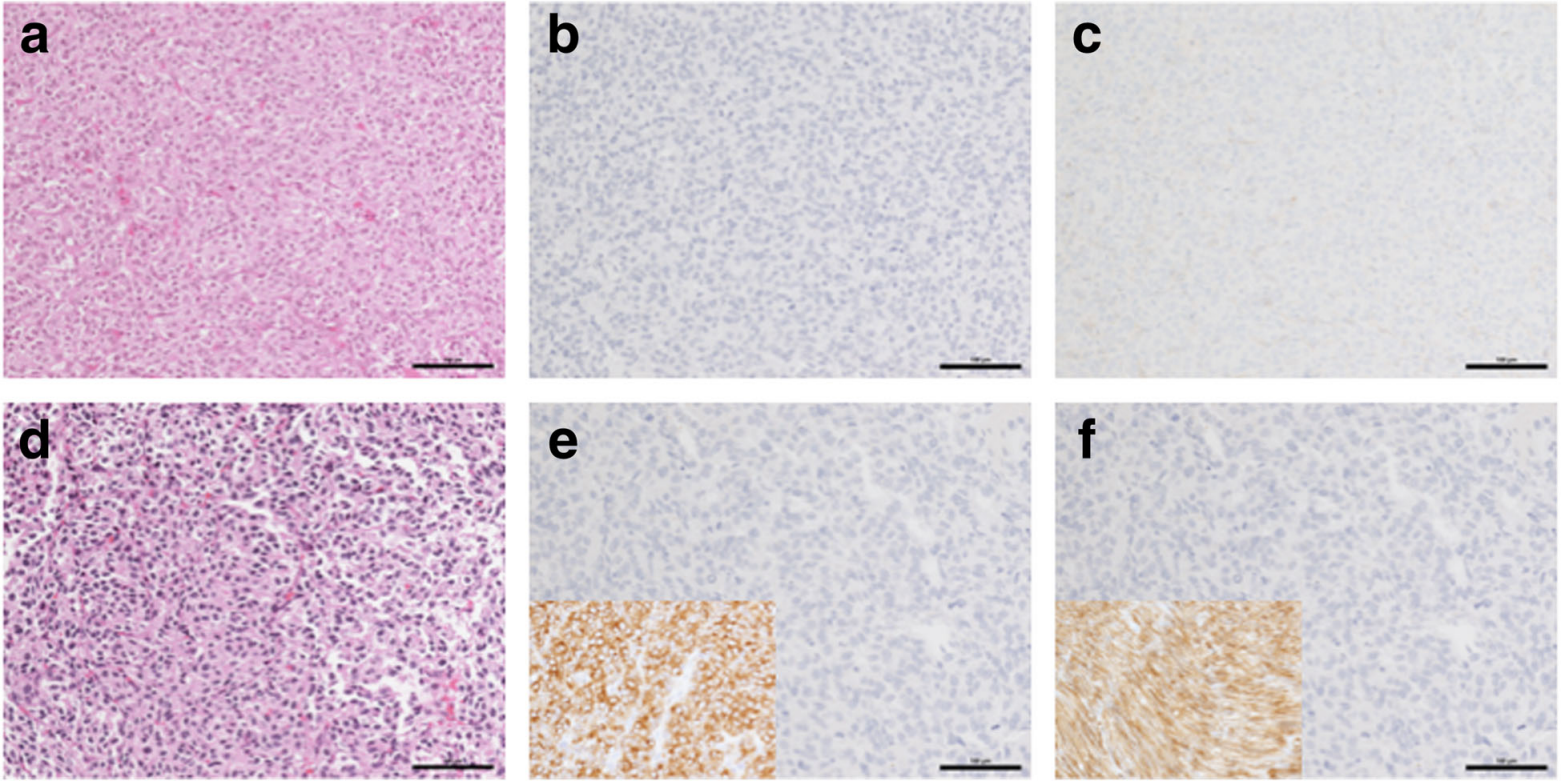
Immunohistochemical analysis of GIST specimens. Panels A, D: H&E for GIST 1 and GIST 2. Panels B, E: CD117 staining for GIST 1 and 2; insert: positive CD117 staining in a control GIST specimen. Panels C, F: DOG1 staining for GIST 1 and 2; insert: positive DOG1 staining in a control GIST specimen

The patient’s primary tumor, an intermediate to high risk, ~10 cm gastric GIST, was resected at an outside institution. Per report, the tumor exhibited weak and focal positivity for CD117 (KIT), vimentin and calretinen, and was negative for SMA, S100, CD34, and desmin. A recurrent GIST was detected approximately 1 year later on surveillance CT, and IM therapy (400 mg daily) was initiated for a period of 3 months. Additional masses were detected in the upper abdomen and left lower quadrant, and progressive disease was confirmed by biopsy. Treatment with sunitinib was initiated (50 mg daily, 4 weeks on 2 weeks off) for 3 cycles. Restaging CT scan again showed disease progression, and sunitinib was discontinued. Subsequently the patient was referred to Fox Chase Cancer Center (FCCC), where a left lower quadrant abdominal wall metastasis was resected and banked by the FCCC Biosample repository (described herein as GIST 1). Direct Sanger sequence analysis of DNA from this tumor by the FCCC Clinical Molecular Genetics Laboratory detected an exon 18 D842V mutation in the *PDGFRA* gene. The patient was enrolled in a Phase II trial (NCT01243346) testing the RTK inhibitor crenolanib in GIST with IM-resistant *PDGFRA* mutations. The patient remained on crenolanib for 3 months until progression. The patient was off trial for a period of ~1.5 months, and again underwent surgery at FCCC for removal of metastatic small bowel and abdominal wall GISTs (GIST 2). Both GIST 1 and GIST 2 retained a high degree of cellularity despite the patient’s history of treatment with targeted therapeutics (Fig. 1a, d). The tumors displayed the epithelioid cell morphology seen in many *PDGFRA*-mutated GISTs, with focal areas displaying a partially developed organoid pattern of cells. Both recurrent tumors were essentially negative for KIT and DOG1 staining (Fig. 1b, e, c, and f). While DOG1 has been shown to be a somewhat more sensitive marker for GIST than KIT and is useful in evaluating KIT-negative tumors, one report has demonstrated that just over 1/3 of KIT-negative GIST stain positive for DOG1 [41], and evaluation of large GIST series for both markers have identified cases, including those harboring mutations in *PDGFRA,* that are both KIT- and DOG1-negative [42, 43].

**Fig. 2:**
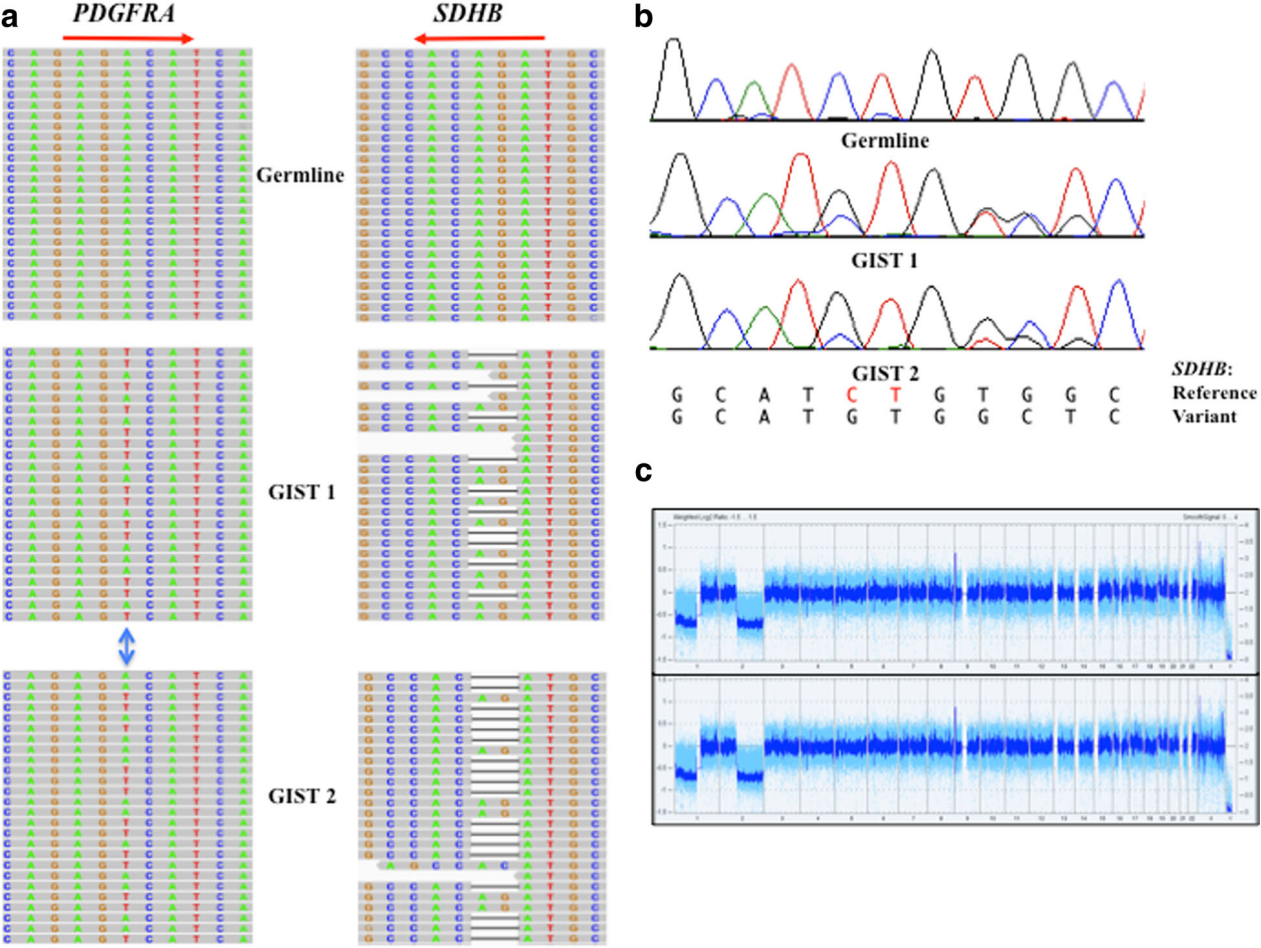
**a** A subset of reads from WES analysis visualized on the Integrative Genomics Viewer (IGV). Left panels show the heterozygous *PDGFRA* c.2525A > T mutation in the two tumors (indicated by *double-headed blue arrow*) that results in the p.D842V amino acid change. Right panels show the two base-pair deletion in exon 4 of the *SDHB* gene (represented as a bar) in reads from both tumors but not in the patient’s germline DNA. Red arrow indicates direction of transcription of *PDGRA* and *SDHB*. **b** Sanger sequencing confirming the frameshift *SDHB* deletion (c.291_292delCT, p.Iso97Metfs*21) in the patient’s GISTs. In the reference sequence the deleted bases are shown in red, and the variant sequence can be seen overlaying the reference sequence in the chromatograms from the tumors. **c** Whole genome view from the Chromosome Analysis Suite (Affymetrix) shows weighted SNP log-2 ratio (*light blue*) and smoothened signal (*dark blue*) for GIST 1 (top panel) and GIST 2 (bottom panel). Large-scale chromosome losses across chromosome arms 1p and 2q can be seen in both tumors

**Fig. 3:**
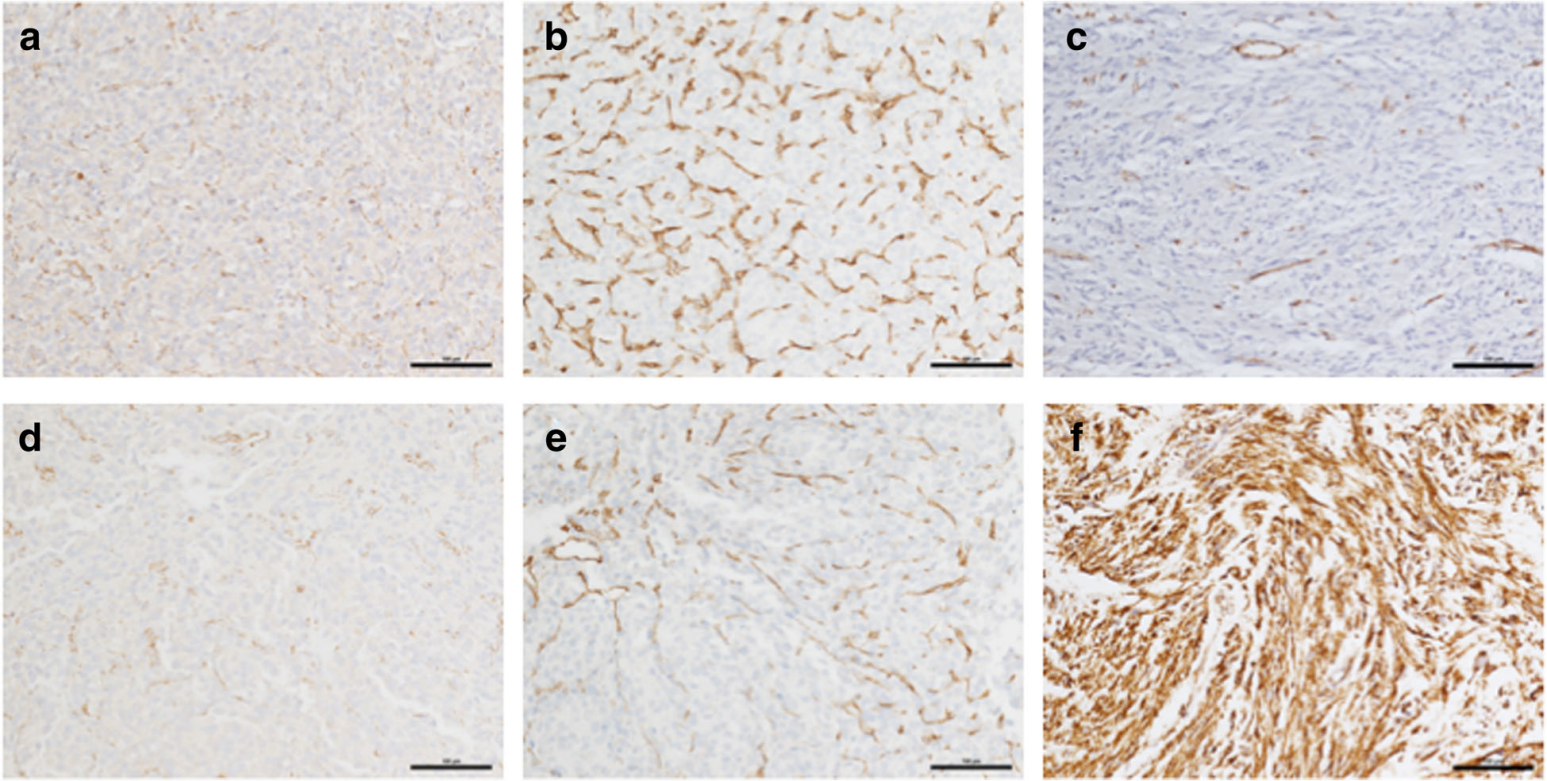
Panels **a**, **d** SDHB IHC for GIST 1 and GIST 2 shows distinct punctate staining in endothelial cells while tumor cells are negative for SDHB. Panels **b**, **e** IHC for endothelial marker CD31 for GIST 1 and GIST 2. Panel **c** Negative SDHB IHC for a control SDH-deficient RTK-wild type GIST. Panel **f** Positive SDHB staining for an SDH-competent *KIT* mutant GIST

Whole exome sequencing (WES) was used to investigate mechanisms of drug resistance in these GISTs. Ten deleterious missense or stop-gain somatic mutations (including the heterozygous *PDGFRA* D842V mutation) were found in both tumors, along with 3 mutations found only in GIST 2 (Fig. 2a, Table 1). A two-base frameshift deletion (c.291_292delCT, p.I97Mfs*21) in exon 4 of the *SDHB* gene was also identified in both GIST 1 and 2 (Fig. 2a). This mutation is predicted to result in a 21 amino acid residue frameshift beginning at residue 97 and ending with a stop codon. The *SDHB* mutant allele frequency from the WES analysis was 42 and 61% in GIST 1 and GIST 2, respectively. Sanger sequencing (Fig. 2b) confirmed the two-base deletion: the chromatograms indicate the wild type allele peaks are also present as a minority population. SNP array analysis (Cytoscan HD, Affymetrix) identified copy number loss across chromosome arm 1p, which encompasses the *SDHB* gene locus, in both tumors (Fig. 2c). This data suggests loss of the wild type *SDHB* allele and loss of SDH function in the tumors. To explore this further, tissue sections for the two tumors were evaluated for expression of SDHB. Although the WES analysis detected wild type reads in the area with the two base *SDHB* deletion, IHC for this subunit indicated that the tumor cells in both specimens were completely negative for SDHB staining (Fig. 3a, d), while endothelial cells surrounding tumor cells displayed strong punctate staining indicative of an intact SDH complex. IHC for the endothelial cell marker CD31 (Fig. 3b, e) indicated the tumors were highly vascularized, suggesting the wild type *SDHB* allele component in the WES and Sanger sequencing may derive from the substantial endothelial cell compartment in the tumors. Our interpretation of the molecular data and IHC analysis for this case is that both GISTs exhibit SDH deficiency in addition to harboring the gain of function PDGFRA mutation.

As *SDHX* and *KIT/PDGFRA* mutations are generally considered to be mutually exclusive oncogenic events in GIST, we explored this phenomenon further by screening a panel of ~75 GIST cases with known genotype status from our institution. In all we analysed 19 *PDGFRA* mutated tumors (including the two from the index case and 10 additional D842V mutants), 50 *KIT* cases, and 5 previously described RTK-wild type SDH-deficient cases [26]. Several representative sections are shown in Fig. 3. While SDHB deficiency was clearly seen in RTK-wild type GIST cases (Fig. 3c shows one example), all *PDGFRA*- or *KIT*-mutated tumors were SDH-competant as indicated by punctate SDHB staining (Fig. 3f).

The WES analysis of GIST 1 and GIST 2 identified somatic mutations in 12 other genes in addition to the *PDGFRA* and *SDHB* mutations (Table 1). Although to our knowledge none of these genes have been implicated in GIST tumorigenesis, ceramide synthase 2 (CERS2) has recently been implicated in breast cancer (reviewed in [44]), while endothelin 3 (EDN3) has been shown to play a role in melanocyte differentiation, and altered endothelin signaling may be involved in melanomagenesis or progression [45].

## Discussion

GIST oncogenesis in the great majority of cases is due to activation of kinase signaling due primarily to gain of function mutations in the KIT and PDGFRA receptors. This class of GIST, which has been referred to as “Type 1” GIST [46], can occur throughout the GI tract, and generally presents as sporadic disease in older adults. Tumor cells are generally spindled or mixed spindled/epithelioid, and these GISTs, with notable exceptions such as the *PDGFRA* D842V mutation discussed here, generally respond well to front-line therapy with the RTK inhibitor IM. SDH-deficient GIST, which have have also been termed Type II GIST, are an interesting sub-class of GIST that generally lack receptor mutations. SDH impairment leads to cytosolic accumulation of the TCA intermediate succinate and inhibition of alpha-ketoglutarate dependent dioxygenases [47]. These enzymes include the prolyl hydroxylase (PHD) that normally contributes to the degradation of the hypoxia-inducible gactor-1A (HIF1A) [48] as well as TET family DNA hydroxylases that are required for demethylation of genomic DNA [49]. Stabilization of HIF1A is thought to lead to induction of transcriptional programs that foster increased angiogenesis and cell proliferation [17], while TET inhibition leads to global DNA hypermethylation that is also likely involved in the oncogenic transformation to GIST [47]. Estimates of SDH deficiency as a percentage of RTK-wild type GIST range from 40 to 85% [2, 33, 50], and more or less distinct sub-groups can be defined based on the molecular and genetic aspects of the defect. Germline mutations in the *SDHB-D* gene subunits are the causative factor in the familial CSS [19], a relatively rare condition which predisposes carriers to the development of gastric GIST and PGL. Mutations in the *SDHA* subunit gene have recently been reported by a number of investigators in apparently sporadic adult cases [26–29, 31, 50, 51]. Finally, epigenetic silencing specific to the *SDHC* gene has been described in syndromic CT cases, a non-familial association of gastric GIST with PGL, pulmonary chondromas and other tumors, as well as in pediatric cases [23–25]. Regardless of the oncogenic insult, SDH-deficient GIST as a class exhibit clinical, pathological, and molecular features that distinguish them from SDH-competent, predominantly RTK-mutant GIST [46]. These tumors almost always present in the stomach, often as multifocal tumors with predominantly epithelioid cell morphology, and have a greater tendency to metastasize to lymph nodes and liver, although these metastases exhibit mostly indolent growth. Patients are generally younger and predominantly female: this is especially true of the *SDHC* epimutant patient class. In a recent analysis of RTK-wild type GIST from the NIH Pediatric and WT GIST Clinic, Boikos et al. reported a median age of 15 y and a female predominance of 95% for patients with epimutant *SDHC* GIST, while the *SDHX* mutant GIST patient demographic was slightly older with less of a female predominance (median age of 23, 62% female) [50]. SDH-deficient GIST also exhibit distinct gene expression patterns, including over-expression of the insulin-like growth factor 1 receptor (IGF1R) [26]. Importantly, due to their distinct oncogenic mechanism these tumors exhibit primary reistance to IM.

The ability to ascertain SDH status readily using IHC for the SDHB subunit has added support to the notion that SDH deficiency in GIST is generally mutually exclusive to the other known oncogenic mechanisms. The initial studies in GIST, focused on syndromic GIST, identified SDH deficiency in CSS-associated GIST as well as GISTs from CT patients, while RTK-mutant GIST or NF1-associated GIST used as controls were SDH-competent [52, 53]. Janeway et al. used a combination of *SDHX* sequencing and SDHB IHC and/or immunoblotting to determine the SDH status of a series of GIST, including 34 sporadic RTK-wild type tumors (pediatric and young adult), 18 *KIT*-mutated tumors, and 5 NF1-associated tumors [54]. Loss of function *SDHB* and *SDHC* gene mutations were identified in 3 young adult and one pediatric RTK-wild type cases, respectively, while an additional 18/18 pediatric cases and 8/12 adult RTK-wild type tumors were deemed SDH-deficient by loss of SDHB protein expression. Only 4 of the SDH-deficient cases were analyzed for *SDHA* mutation, possibly accounting for the low percentage of *SDHX* gene mutation in this group (13%). Notably, all 5 NF1-associated GIST and 17/18 of the *KIT*-mutated GIST analyzed were SDH-competent. Due to the study’s focus on RTK-wild type GIST, the manuscript contains no further details on the *KIT* mutant, SDH-deficient case. The Miettinen group examined a series of 756 gastric GIST by SDHB IHC and identified 66 cases of SDH-deficient GIST [32]. In contrast, the 378 non-gastric GISTs they analyzed were all found to be SDH-competent. The 66 gastric SDH-deficient GIST were all found to be wild type for *KIT*, *PDGFRA*, and *BRAF.* The authors failed to detect mutations in *SDHX* genes, although a limited number of exons in the *SDHB-D* genes were covered, and *SDHA* was not analyzed*.* In a study focusing on NF1-associated, RTK-wild type GIST, Wang et al. failed to detect SDH deficiency in all 22 tumors analyzed, confirming previous studies that looked at smaller NF1 patient sets [55]. Doyle et al. examined SDHB expression in a large cohort of GIST with known *KIT/PDGFRA* mutational status (*n* = 264) [33]. Among 53 RTK-wild type GIST they identified 22 SDH-deficient tumors (42%), of which all 21 with known anatomical location originated in the stomach. In this study all RTK-mutant GIST examined (179 *KIT*, 32 *PDGFR*) were immunohistochemically positive for SDHB expression.

These large series confirmed that SDH deficiency is found in a subset of RTK-wild type gastric GIST cases, whereas *KIT*/*PDGFRA*-mutated and NF1-related GISTs are generally SDH-competent. Against this background we searched the GIST literature for cases such as ours that documented SDH deficiency in RTK-mutant GIST. The search identified three case studies involving CSS patients with germline *SDHX* mutations and somatic *KIT* mutations in their GIST, as well as several apparently sporadic cases. Ayala-Ramirez et al. presented a case of a 33 year old male patient with bilateral pheochromocytoma (PCC), an extra-adrenal PGL and a gastric GIST [56]. Although there was no family history of PCC/PGL, genetic analysis identified a germline truncating mutation in the *SDHD* gene (p.W43*), supporting the diagnosis of CSS. Genotyping of the patient’s GIST revealed a known gain of function *KIT* mutation (p.D579del) [57]. However, SDHB IHC was not performed, and there was no *SDHD* genetic analysis described for the GIST, so it is difficult to say if the tumor was SDH-deficient. Gasparotto et al. described a 38 year old female with CSS with a high risk spindle cell rectal GIST and two cervical bilateral PGLs [58]. They identified an activating *KIT* mutation in the GIST (p.W557_V559delinsF) along with an *SDHD* frameshift mutation mutation (p.C150Yfs*42). However, the *SDHD* mutation was heterozygous and the authors report weak focal SDH staining in the GIST, while completely absent in the PGL. In addition, the rectal location and spindle cell morphology are not characteristic of SDH-deficient GIST. These two reports leave open the possibility that the cases described were sporadic KIT driven GISTs that occurred on a genetic background of heterozygous germline *SDHD* mutations. Jove et al. present a case report on a CSS patient that more robustly documents the co-occurrence of two oncogenic mechanisms in the same GIST [59]. The male patient was first treated surgically for a PGL at the age of 13; a heterozygous *SDHB* deletion (c.166_170del5, p.P56Yfs*5) was identified from peripheral blood [60]. Subsequently at age 29, the patient underwent resection of a multinodular gastric GIST that harbored a heterozygous *KIT* mutation (p.L576P), previously described as an activating mutation in GIST [61]. SDHB IHC for the GIST was completely negative, with positive staining in internal control cells. Adding strength to the analysis, sequencing of the patient’s tumor DNA demonstrated LOH of the wild type *SDHB* allele. Two relevant cases were identified in a larger study of 95 RTK-wild type GIST reported by Boikos et al. [50]. Among their subset of SDH-deficient, *SDHX* mutated GIST, the case referred to as GIST 077 harbored a germline stop-gain *SDHA* mutation (p.R512X) and somatic loss of the wild type allele, as well as a gain of function mutation in KIT (p.L576P). The second case, GIST 117, carried a germline *SDHB* splice site mutation (c.423 + 1G > A) and exhibited loss of the wild type allele in the tumor. This tumor also contained an activating KRAS alteration (p.G12D), a mutation that was also described in several *KIT* mutant GIST cases in a single report [16]. Finally, Ondrej and colleagues described a case of 52 year old male patient with a large mass in the posterior mediastinum, originally diagnosed as pleomorphic rhabdomyosarcoma, who subsequently underwent resection for multiple recurring nodules in the greater omentum [62]. Histological diagnosis of GIST was supported by strong CD117 staining and the identification of an activating *KIT* mutation (p.W557_K558del) in the tumor tissue. The authors also identified a missense variation in the *SDHD* gene (p.G12S), and IHC for the SDHB subunit showed faint and focal staining in the tumor tissue as compared to endothelial cells serving as internal controls. Although this GIST would be an unusual example of an extra-gastric SDH-deficient GIST also harboring an oncogenic *KIT* mutation, the G12S variant identified may represent an *SDHD* polymorphism as opposed to an inactivating mutation, as it was previously identified in 8/200 (4%) of healthy individuals [63].

Our literature search did not identify other examples of *PDGFRA* mutant GIST that also exhibited SDH deficiency. The tumors we examined in our case report were separate recurrences approximately 3 years after resection of the primary gastric tumor. Both tumors had a heterozygous *PDGFRA* D842V mutation (Fig. 2), which has been shown to confer clinical resistance to IM [7, 9] and sunitinib [64]: indeed the patient showed rapid disease progression on these two drugs (~3 months and 5 months, respectively). The patient’s GISTs also exhibited SDH deficiency due to a somatic frameshift mutation in the *SDHB* subunit gene and loss of the wild type allele (Figs. 2 and 3). Patients with RTK-wild type GIST (including SDH-deficient as well as SDH-competent GIST) have lower IM response rates compared to *KIT* exon 11 mutant GIST [9, 10], although sunitinib response rates have been higher for RTK-wild type GIST [64]. Theoretically, either mutation could be a driver in GIST, however the patient’s primary tumor was not available for analysis. GIST oncogenesesis by PDGFRA activation requires only a single hit, and the D842V mutation accounts for oncogenesis in about 1:20 GIST cases [2]. Oncogenesis through *SDHX* mutations occurs less frequently and requires inactivation of both alleles (one of which is usually due to a germline mutation), so the SDH inactivation may have been a later event. Interestingly, the patient also progressed rapidly (~3 months) on the investigational PDGFRA inhibitor crenolanib, which has been shown in biochemical and cellular models to be ~100–150-fold more potent than IM against the D842V mutation [65]. It is tempting to speculate that SDH deficiency in the tumor may have contributed to the lack of response to this selective PDGFRA inhibitor.

Although the identification of an activating mutation in either *KIT* or *PDGFRA* supports the clinical diagnosis of GIST and can be used to guide therapy, it does not preclude the possibility of the presence of *SDHX* gene mutations in these tumors, suggesting that the inclusion of these genes may be warranted in routine testing for GISTs. As detailed above, reports that clearly document GISTs with kinase and *SDH*X mutations are rare but not non-existent. Most documented cases are either CSS patients with both GIST and PGL and/or PCC tumors, or carry the germline *SDHX* mutations that predispose to the development of these tumors. Our case is unique in that both oncogenic mutations (*PDGFRA* and *SDHB*) were somatic and there was no germline involvement. Patients with germline *SDHX* mutations require long-term screening for disease detection and management [56] as well as genetic counseling and/or testing of family members. For GIST patients that carry germline *SDHX* mutations along with a *KIT* or *PDGFRA* mutation, standard IM therapy may still be effective. Anecdotally, in the CSS case described by Gasparotto et al. [58], IM treatment targeted to the exon 11 *KIT* mutation led to significant response and tumor stabilization for the patient. Further investigation is required to understand the biological and clinical consequences of SDH deficiency in GISTs with activating *KIT* or *PDGFRA* gene mutations. While molecularly targeted treatment options tailored to SDH deficiency in GIST are limited, a recently completed phase 2 clinical trial of linsitinib, an inhibitor of the IGF1 receptor that is over-expressed in RTK-wild type GIST, indicated that the drug provided clinical benefit in 45% of patients, albeit without RECIST response [66]. As mentioned above, sunitinib as well as regorafenib have had some success in these tumors [64, 67], possibly due to their ability to target the vascular endothelial growth factor receptor (VEGFR) and angiogenesis [68]. Finally, the global DNA hypermethylation seen in all SDH-deficient tumors, or the specific epigenetic silencing of *SDHC* seen in the CT/pediatric subset, suggests the potential of demethylating agents such as decitabine for treating these GISTs [24, 47].

## Conclusions

We report a unique case of an SDH-deficient GIST case with an activating *PDGFRA* mutation. Oncogenic mutations in GIST are generally mutually exclusive; however documented exceptions exist which may have diagnostic and therapeutic implications.

## Additional file

Additional file 1: Primers for Sanger sequencing. (DOCX 14 kb)

## Abbreviations

CSS: Carney-Stratakis syndrome
CT: Carney triad
CT: Computed tomography
GIST: Gastrointestinal stromal tumor
HIF: Hypoxia inducible factor
ICC: Interstitial cells of Cajal
IGF1R: Insulin-like growth factor 1 receptor
IHC: Immunohistochemistry
IM: Imatinib mesylate
NF1: Neurofibromatosis type I
PC: Pulmonary chondroma
PCC: Pheochromocytoma
PDGFRA: platelet derived growth factor receptor alpha
PGL: Paraganglioma
PHD: Prolyl hydroxylase
RTK: Receptor tyrosine kinase
SDH: Succinate dehydrogenase
SNP: Single nucleotide polymorphism
TMA: Tissue microarray
WES: Whole exome sequencing
