# Supplementary material for: Succinate dehydrogenase deficiency in a PDGFRA mutated GIST
Source: BMC Cancer. 2017 Aug 2;17:512. doi: 10.1186/s12885-017-3499-7 (PMC5541693; doi:10.1186/s12885-017-3499-7)
Supplement: Additional file 1: — Primers for Sanger sequencing. (DOCX 14 kb) [file 12885_2017_3499_MOESM1_ESM.docx]

| Additional File 1. Primers for Sanger sequencing. | |
| --- | --- |
| Gene exon-orientation | Sequence (5’-> 3’) |
| *PDGFRA* 18-F | ACCATGGATCAGCCATGCTT |
| *PDGFRA* 18-R | TGAAGGAGGATGAGCCTGACC |
| *SDHB* 4-F | ATGTTGCATGTCAGTGCTGC |
| *SDHB* 4-R | ACTCAAACAAATCCTGCCCTG |
| *CERS2* 9-F | AAGGAGGAGGGCACAGAGTC |
| *CERS2* 9-R | AGGCAGGATAGAGCTCCAGTG |
| *DNAH3* 34-F | ATATTGGGAATGTCTGCTGGG |
| *DNAH3* 34-R | AAGAATGGAAGATGGAAGAGGG |
| *CAPN9* 16-F | TCACCCATTTACTGCCATCAG |
| *CAPN9* 16-R | GTGTCAGGAAGTGAGTCCTGG |
| *DIS3* 8-F | ATTGTGGCATGCTTTCCAAG |
| *DIS3* 8-R | CATGCTACTTACCAGGTCACTTTG |
| *GJD2* 2-F | GTTCCGAAATGCCCTGGAAA |
| *GJD2* 2-R | TTGAGCACAACACAGATGCC |
| *EDN3* 2-F | CCCTCCTCAGGTGTTTGGG |
| *EDN3* 2-R | GTCCTCCTGGGAATGAGCAG |
| *PI4KA* 28-F | GGGAGACATAGTTCAGCCCA |
| *PI4KA* 28-R | GGACTTGGTGACGGTAGGTG |
| *TENM2* 23-F | GCGACTGATGCCATCTTGAA |
| *TENM2* 23-R | GGCTCACAGTGTATTGGTGG |
| *JPH1* 3-F | GTCAGATTTGACCTTGGGCAG |
| *JPH1* 3-R | TTGCTCGAGATGGAAGACTTG |
| *DIS3L2* 2-F | AGAGTTGATTGCTGCCTCCT |
| *DIS3L2* 2-R | AGCACCTGGATGAGTGTTCC |
| *SENP6* 7-F | TATGTGGAGATCCTGCTGAGG |
| *SENP6* 7-R | GCAGGGAAGCACTGATAAATTC |
| *PLCG2* 18-F | CTAGGAGCAGAGGGAAGGTTG |
| *PLCG2* 18-R | TTTGGAAACTGGCCACCTC |
